# Resolving cryptic species complexes in marine protists: phylogenetic haplotype networks meet global DNA metabarcoding datasets

**DOI:** 10.1038/s41396-021-00895-0

**Published:** 2021-02-15

**Authors:** Daniele De Luca, Roberta Piredda, Diana Sarno, Wiebe H.C.F. Kooistra

**Affiliations:** 1 Department of Integrative Marine Ecology, Stazione Zoologica Anton Dohrn, Naples, Italy; 2 Department of Research Infrastructure for Marine Biological Resources, Stazione Zoologica Anton Dohrn, Naples, Italy; 3 Present Address: Department of Biology, Botanical Garden of Naples, University of Naples Federico II, Naples, Italy

**Keywords:** Population genetics, Biodiversity, Biogeography, Next-generation sequencing

## Abstract

Marine protists have traditionally been assumed to be lowly diverse and cosmopolitan. Yet, several recent studies have shown that many protist species actually consist of cryptic complexes of species whose members are often restricted to particular biogeographic regions. Nonetheless, detection of cryptic species is usually hampered by sampling coverage and application of methods (e.g. phylogenetic trees) that are not well suited to identify relatively recent divergence and ongoing gene flow. In this paper, we show how these issues can be overcome by inferring phylogenetic haplotype networks from global metabarcoding datasets. We use the *Chaetoceros curvisetus* (Bacillariophyta) species complex as study case. Using two complementary metabarcoding datasets (Ocean Sampling Day and Tara Oceans), we equally resolve the cryptic complex in terms of number of inferred species. We detect new hypothetical species in both datasets. Gene flow between most of species is absent, but no barcoding gap exists. Some species have restricted distribution patterns whereas others are widely distributed. Closely related taxa occupy contrasting biogeographic regions, suggesting that geographic and ecological differentiation drive speciation. In conclusion, we show the potential of the analysis of metabarcoding data with evolutionary approaches for systematic and phylogeographic studies of marine protists.

## Introduction

The term ‘cryptic species’ is used for morphologically indistinguishable taxa for which there is evidence (genetic, ecological, behavioural, biological, etc.) that they belong to different evolutionary lineages [1, 2]. Groups of such taxa are commonly referred to as ‘cryptic species complexes.’ Cryptic species may have diverged recently and not yet have become morphologically distinct, or they are distantly related but retained their ancestral morphology or converged morphologically [3, 4].

Recent molecular taxonomic studies have uncovered remarkably high cryptic species diversity in marine planktonic protists [5–7]. Originally such taxa were believed to be lowly diverse because potentially high dispersal opportunities in the open sea leave little opportunity for genetic differentiation even at a global scale [8–12]. Unfortunately, exploring geographic patterning of cryptic species by traditional means, i.e. of sampling and examining large numbers of specimens from all over the oceans, is a daunting task. The few truly global biogeographic studies of such species complexes to date [13, 14] reveal that the individual species can be cosmopolitans in their own right or be geographically more confined.

Exploration of species distribution patterns in marine planktonic protists requires accurate species delimitation [15, 16]. Cryptic species complexes in protists are usually explored by comparing nucleotide differences on selected DNA markers with differences in the ultrastructural, biochemical, biological or ecological properties of selected strains [13, 16–18]. Classically, nucleotide data are gathered through Sanger sequencing [19–21]. If genetic distances or bootstrap values justify independent evolutionary lineages, cryptic species are hypothesised. Yet, phylogenies depict sharply bifurcating speciation events and vertical changes within ancestor-descent lineages [22], but in case of recent speciation, attenuated gene flow may still persist between the sister lineages, which is visualised better in phylogenetic networks [22–24].

In recent years, high throughput sequencing of taxonomically discriminative barcode regions (HTS metabarcoding) has revolutionised our capacity to explore protistan diversity in environmental DNA samples [25]. However, finding a single, universal DNA barcode for a genetically heterogeneous assemblage like protists has revealed to be virtually impossible because of their long, independent, and complex evolutionary histories [26]. Several protistan DNA barcodes have been proposed, such as the D1–D2 or D2–D3 regions of the 28S rRNA gene [27–29], the ribosomal internal transcribed spacers ITS1 and ITS2 [30–32], the mitochondrial gene COI [33] or the chloroplastic gene *rbc*L [34], but the ~500 bp variable V4 region of the 18S rRNA gene has been preferred as the universal protistan barcode [26]. It is part of a multi-copy region present in all eukaryotes and includes conserved and variable regions useful for recognition at various taxonomic levels. The 18S rRNA gene is used extensively to infer phylogenies and, consequently, reference sequences are available for a comprehensive set of species from across the protistan diversity [26, 35, 36]. The resolution of the 18S rRNA gene to distinguish species has been positively tested in several protistan taxa as foraminifera [37], dinoflagellates [38] and some diatoms [35, 39], but also negative results have been reported [26, 40]. Global metabarcoding initiatives targeting marine protistan diversity used variable regions in the 18S rRNA gene as metabarcode marker; Ocean Sampling Day 2014 (OSD) [41] selected the V4 variable region whereas Tara Oceans [42] used the shorter V9variable region. The resulting datasets have been used to uncover protistan diversity and distribution [43–46], to analyse their phylogenetic relationships [47, 48] and to delimit species [49, 50].

**Fig. 1:**
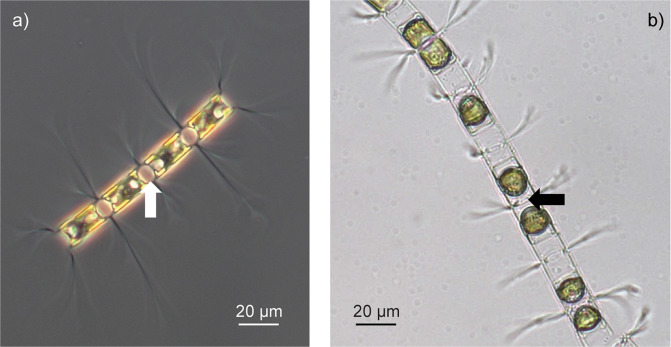
Light microscopy photographs of *Chaetoceros curvisetus* and *C. pseudocurvisetus*. **a** *C. curvisetus*; **b** *C. pseudocurvisetus*. The size and shape of aperture between sibling cells (see arrows) are useful characters for distinguishing these taxa.

In the present study, we aim at delimiting species in the *Chaetoceros curvisetus* (*C. curvisetus*) species complex and at mapping their distribution patterns. The complex belongs to the diatoms, a diverse class of unicellular algae abundant in marine and freshwater habitats. *Chaetoceros* is arguably the most abundant and diverse genus in the marine planktonic diatoms. Its hallmark is constituted by setae—tubular silica extensions extending from the frustule and linking cells into chains. To date, only two species have been described: *C. curvisetus* Cleve and *C. pseudocurvisetus* Mangin (Fig. 1) [51]. Recent taxonomic studies uncovered that *C. curvisetus* consists of several genetically distinct taxa [39, 52, 53] with *C. pseudocurvisetus* resolved amongst these taxa, rendering the morphospecies *C. curvisetus* sensu lato paraphyletic.

Here we use the molecular information contained in the metabarcode data of OSD [41] and Tara Oceans, coupled with a phylogenetic network approach to identify cryptic species and assess their phylogenetic relationships. Reference sequences of the 18S rRNA gene of *C. curvisetus* species and close outgroup taxa [39] are used to gather reads putatively belonging to the species complex from these datasets. The extracted reads are sorted into haplotypes, which are used to generate phylogenetic networks. From the latter we delineate the species within the complex, explore the evolutionary relationships and possible gene flow among the species and assess their phylogeographic distribution and abundance in Longhurst’s biogeographic provinces [54].

## Materials and methods

### Reference molecular dataset

The reference dataset comprised ten 18S rRNA genes obtained from cultured strains, and retrieved from NCBI (Table 1). All the ten 18S rRNA gene references included the V4 region as amplified by the primers used in OSD [36], modified from [55]), whereas two of them did not cover the V9 region as amplified by the Tara Oceans primers ([56]; see Table 1). To simplify the nomenclature of the species belonging to this complex (some genetically defined previously [39, 53], others identified in the present study) we indicated the taxa in the *C. curvisetus* complex as sp. 1–sp. 11 (Table 2). From the full-length 18S rRNA gene sequences we extracted the V4 and V9 regions, corresponding with the fragments amplified in OSD and Tara Oceans, respectively. None of the ‘curvisetus’ species shared identical V4 and V9 barcodes.

**Table 1:** List of 18S rRNA gene references for the V4 and V9 regions utilised for gathering taxa in the *C. curvisetus* species complex.

Taxon	Strain	GenBank accession number
*C. curvisetus* SKLMP	YG033	MG821562, V9 not included
*C. curvisetus* 1	Na10C1	MG972232
*C. curvisetus* 2	Na1C1	MG972235
*C. curvisetus* 2c	El6A2	LC466961
*C. curvisetus* 3	newBB2	MG972241
*C. curvisetus* 3e	El4A2	LC466962
*C. pseudocurvisetus*	IRB	MG385841, V9 not included
*C. pseudocurvisetus*	Na13C4	MG972304
*C*. cf. *tortissimus*	Na18C4	MG972275
*C. tortissimus*	Na25A2	MG972325

**Table 2:** List of species abbreviations utilised in the present study.

Species (this study)	Abbreviation (this study)	Corresponding species	Reference for the species
*C*. sp. 1	sp. 1	*C. curvisetus* 1	[39, 52]
*C*. sp. 2	sp. 2	*C. curvisetus* 2	[39, 52]
*C*. sp. 3	sp. 3	*C. curvisetus* 3	[39]
*C*. sp. 4	sp. 4	*C. curvisetus* 2c	[53]
*C*. sp. 5	sp. 5	*C. curvisetus* 3e	[53]
*C*. sp. 6	sp. 6	*C. pseudocurvisetus*	[39, 52]
*C*. sp. 7	sp. 7	*C. curvisetus* SKLMP YG033	[GenBank direct submission]
*C*. sp. 8	sp. 8	Putative new species (OSD dataset)	[This study]
*C*. sp. 9	sp. 9	Putative new species (OSD dataset)	[This study]
*C*. sp. 10	sp. 10	Putative new species (Tara dataset)	[This study]
*C*. sp. 11	sp. 11	Putative new species (Tara dataset)	[This study]

### Downloading and processing of metabarcode data

The OSD dataset included 144 samples for the V4 region of the 18S rRNA gene (https://mb3is.megx.net/osd-files?path=/2014/datasets/workable) and 31 samples for the V9 region of the 18S rRNA gene (https://mb3is.megx.net/osd-files?path=%2F2014%2Fdatasets%2Fworkable%2Frdna). From the V4-OSD workable fasta files, we generated a total fasta file with the unique haplotypes and a table containing the abundance of their reads at each site (Total OSD abundance table) using mothur v1.41.1 [57]. In this manuscript, we indicate with the term ‘haplotype’ a group of identical cleaned reads. The same procedure was done for OSD-V9 workable fasta files. The Tara Oceans V9 dataset was downloaded from https://doi.pangaea.de/10.1594/PANGAEA.873277 and ENA (accession number: PRJEB9737; [58, 59]). We directly extracted the fasta file of haplotypes (unique cleaned reads) and the Total Tara Oceans abundance table from the downloaded file containing reads of Tara Oceans’ 210 sites. We then generated a full V9 dataset including OSD-V9 and Tara Oceans data.

### Recovery of species from global metabarcoding datasets

The V4 and V9 fragments of the ten references were used as queries for a local blastn BLAST [60] against OSD (V4) and OSD-V9 + Tara Oceans datasets, respectively, to extract haplotypes at ≥95% similarity. The strategy of using references of close outgroups and a relaxed similarity threshold (95%) ensured inclusion of all haplotypes belonging to the *C. curvisetus* species complex, plus those of unknown species therein. The extracted metabarcode haplotypes were aligned with the ten references using MAFFT online [61] and a phylogenetic tree was built in FastTree v2.1.8 [62], using the GTR model. The resulting tree was visualised and modified in Archaeopteryx v0.9901 [63] to remove haplotypes resolving within outgroup clades (false-positives). This procedure was carried out separately for V4 and V9 fragments. The retained (validated) haplotypes were considered to belong to taxa in the *C. curvisetus* species complex. The abundance and distribution of V4 and V9 *curvisetus* haplotypes were extracted from the Total OSD and Tara Oceans abundance tables. At the end of the validation procedures, four files were generated, containing the validated OSD and OSD-V9 + Tara Oceans sequences in fasta format and the respective abundance tables (available on figshare, see ‘Data availability’ section).

### Phylogenetic haplotype network inference

Phylogenetic haplotype networks were constructed using the statistical parsimony algorithm by [64] implemented in TCS network [65]. Networks were visualised in PopART v1.7 [66] including the information of read abundances for each haplotype. Each network was exported in nexus format and as table containing the list of sequence ID’s (both reference and metabarcode haplotypes) grouped in each node in the network. Considering that the number of mismatches between nodes was normally distributed in both V4 and V9 networks (mean (µ) ± standard deviation (σ), median, and mode for V4: 1.14 ± 0.60, 1, and 1, and for V9: 1.03 ± 0.49, 1, and 1, respectively), we considered 2 mismatches (µ + 2σ), corresponding to the area describing the 95.46% of mismatch distribution, as threshold for errors and intra-specific variation. Based on these assumptions, we inferred species using the following criteria: (1) nodes without the reference and exhibiting ≤2 mismatches with the node containing the reference were attributed to that taxon; (2) nodes without reference and with >2 mismatches with respect to the nodes with reference were considered as hypothetical new taxa if their read-abundance was ≥2. After species inference, we took the representative sequence (the most abundant) of each delimited species and inferred a phylogenetic tree (for V4 and V9 regions) for a rapid and supported visualisation of phylogenetic relationships among taxa. Maximum Likelihood (ML) trees were inferred using IQ-TREE v1.6.8 [67] under the TN + F + G4 model for V4 and the K2P + G4 model for V9 (suggested by ModelFinder, [68]) and 1 000 bootstrap replicates for both datasets. Sequences of *C. tortissimus* and *C*. cf. *tortissimus* were used as outgroup.

### Genetic divergence among species and variability within species

To quantify the relatedness of each species in terms of distances, we calculated the net genetic distance between pairs of species as implemented in MEGA6 [69]. We used the Jukes and Cantor model of sequence evolution [70] to calculate the distances across all metabarcode haplotypes of each species, which best fitted our data. We also calculated, using the same model, the minimum, maximum and average evolutionary divergence of sequences within nodes (the number of base substitutions per site from averaging over all sequence pairs within each group) using MEGA6 [69]. The presence of barcoding gap in the inferred species was explored. The barcoding gap was considered to occur if the maximum distance among sequences within species was lower than the minimum distance among sequences between species [71].

### Global phylogeography of taxa belonging to the *C. curvisetus* species complex

The distribution of the inferred species of the *C. curvisetus* complex was mapped over the world’s oceans. First, from the abundance tables previously generated (see ‘Data availability’ section), we summed the abundances of the haplotypes belonging to the same inferred species. Then, data were normalised to the total number of reads for each sample and reported as percentage. Finally, we plotted the transformed abundance of each inferred species in Longhurst’s provinces in the form of heatmaps. For heatmap generation, we used the R [72] working packages *phyloseq* [73] and *ggplot2* [74]. We also plotted the occurrence of each species over the sample stations on a world map using the packages *maps* [75] and *ggplot2*.

## Results

### Validation of candidate haplotypes in the *C. curvisetus* species complex

**Fig. 2:**
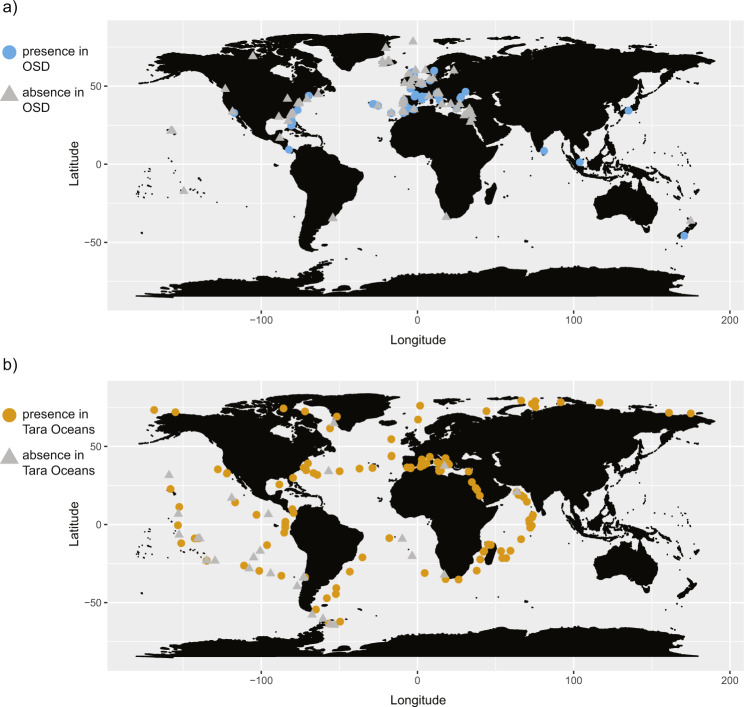
Occurrence of taxa belonging to the *C. curvisetus* species complex. **a** OSD data; **b** Tara Oceans data. Light blue dots refer to occurrence in OSD data (V4 + V9 regions of the 18S rRNA gene), whilst orange dots, in Tara Oceans data. Grey triangles indicate absence in the molecular data from the respective sampling site.

BLAST analysis of the ten reference V4 rRNA gene sequences against the OSD-V4 data retrieved 4223 reads corresponding to 1428 unique (non-redundant) haplotypes. The phylogenetic tree-approach resulted in the validation of 1232 of these haplotypes (3804 reads) as members of the *C. curvisetus* species complex (files V4_OSD_curvi_validated.fasta and OSD_plus_OSD-V9_Tara_abundance_tables.xlsx available on figshare, see Data availability). BLAST analysis of the eight reference V9 rRNA gene sequences against the OSD-V9 and Tara Oceans data returned 2247 haplotypes (856967 reads in Tara Oceans and 194 in OSD-V9). After validation, 772 haplotypes (68210 reads in Tara Oceans and 192 in OSD-V9) were found to belong to the complex (files V9_TARA_LW_curvi_validated.fasta and OSD_plus_OSD-V9_Tara_abundance_tables.xlsx available on figshare, see ‘Data availability’ section). Reads of validated haplotypes were found in 60 out of 144 OSD sampling sites (41.7%) and 117 out of 210 Tara Oceans stations (55.7%) (Fig. 2, Supplementary Table 1).

### Phylogenetic haplotype networks

**Fig. 3:**
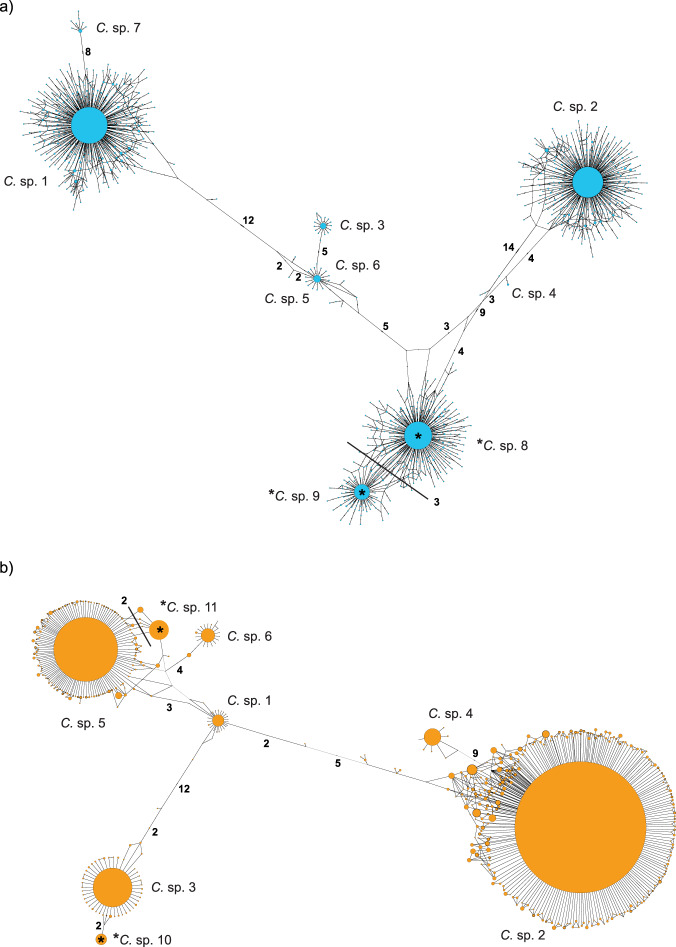
TCS haplotype networks for the *C. curvisetus* species complex. **a** OSD data; **b** OSD-V9 + Tara Oceans data. The size of the nodes refers to the abundance of the reads. Asterisk (*) indicates nodes without a reference barcode corresponding to putative new species. Numbers in bold indicate the number of mutations. Edges with the same number of mutations are marked with a straight line.

The haplotype network based on the OSD dataset (V4 region of the 18S rRNA gene) contained seven nodes assigned to known species in the *C. curvisetus* species complex plus two without a reference (Fig. 3a). Most of the metabarcodes were assigned to sp. 1, 2, 3, 6 and 7 (Fig. 3a). Only one haplotype was recovered for the reference of sp. 5 and, likewise, only one haplotype for that of sp. 4. Many haplotypes clustered into two closely related nodes (spp. 8 and 9) lacking references (Fig. 3a). Moreover, sp. 3 is more closely related to sp. 6 (*C. pseudocurvisetus*) than to the other ‘*C. curvisetus*’ species; sp. 5 (Red Sea) is closely related to sp. 6 and distantly to sp. 1. This latter node is separated by eight mismatches from the reference of sp. 7 from Hong Kong.

The haplotype network based on the Tara Oceans dataset (V9 region of the 18S rRNA gene) contained six nodes assigned to a known species plus two without a reference (Fig. 3b). Most of the haplotypes were assigned to sp. 2; and many to spp. 3 and 5, whereas all the other species were less abundant. The node of sp. 3 was close but clearly separated from a peripheral node with a large number of reads. The same was observed for sp. 5. These peripheral nodes were treated as distinct species (spp. 10 and 11, respectively). Nodes containing the V9 reference sequences of two strains from the Red Sea (spp. 4 and 5) showed high abundance in the Tara Oceans dataset.

The V4 regions of the 18S rRNA gene sequences of spp. 8 and 9, and the V9 regions of the 18S rRNA gene sequences of spp. 10 and 11 were blasted against NCBI GenBank with the aim of retrieving complete 18S rRNA gene sequences showing exact match with the V4 of sp. 8 or 9 as well as the V9 of sp. 10 or 11. Such a finding would demonstrate that nodes in the V4- and V9 networks represent the same species. However, such a connection could not be made because none of these sequences obtained an exact hit. The representative sequences (dominant haplotypes) for these four putative new species are available in GenBank (see ‘Data availability’ section). An alignment of the representative V4 and V9 sequences of each species with sequence signatures is provided in Supplementary Information (Supplementary Fig. 1).

In the network inferred from the V4 region of the 18S rRNA gene (Fig. 3a), the group encompassing spp. 3, 5 and 6 was recovered midway between sp. 1 on one side and spp. 2, 8 and 9 on the other side. Likewise, sp. 4 was recovered in between sp. 2 and spp. 8 and 9. The main branches connecting the nodes showed little reticulation, suggesting reduced gene flow or none at all. The link between sp. 8 and sp. 9 was reticulated, suggestive for gene flow between the two. In the network inferred from the V9 region of the 18S rRNA gene (Fig. 3b), the node attributed to sp. 1 was like a pivot with three branches: one branch with sp. 3, another branch with spp. 2 and 4, and a third branch with spp. 5 and 6. These three branches were devoid of intricate reticulations, suggesting paucity or absence of gene flow.

The ML tree inferred using the V4 representative sequences (the most abundant) of each newly identified putative species plus the references confirmed that the taxa without reference barcodes (spp. 8 and 9) are members of the *C. curvisetus* species complex and likely constitute at least one new species (Supplementary Fig. 2a). The two taxa are closely related and share a common ancestor with sp. 2 (Supplementary Fig. 2a). In this tree, the clade with spp. 1 and 7 was the first to branch off. Instead, in the V9 tree (Supplementary Fig. 2b) the clade with spp. 2 and 4 was sister to a polytomy with the remainder of the ingroup references plus spp. 10 and 11. The haplotype of sp. 10 was recovered as close sister of sp. 3.

### Genetic differentiation and variability

Pairwise genetic distances among the inferred species differed between the V4 and V9 regions of the rRNA gene, but the proportions were comparable. For V4, the lowest inter-specific genetic distances were between spp. 5 and 6 (0.007) and between spp. 8 and 9 (0.008, Supplementary Table 2a), whilst the highest values were observed between spp. 1 and 2 (0.107) and between spp. 2 and 7 (0.105) (Supplementary Table 2a). For V9, inter-specific distances ranged from 0.368 (between spp. 3 and 4) to 0.022 (between spp. 5 and 11) (Supplementary Table 2b). The highest intra-specific distance for the V4 and the V9 regions (0.105 and 0.049, respectively) was higher than their minimum inter-specific distance (0.007 and 0.022, respectively). Therefore, no threshold value could be established to distinguish between inter- and intra-specific variability (barcoding gap). Within each species, the mean evolutionary divergence over sequence pairs ranged from 0.000 (sp. 4) to 0.055 (sp. 5) for V4 region and from 0.000 (sp. 3) to 0.017 (sp. 2) for V9 (Supplementary Table 3).

### Global distribution of taxa belonging to the *C. curvisetus* species complex

**Fig. 4:**
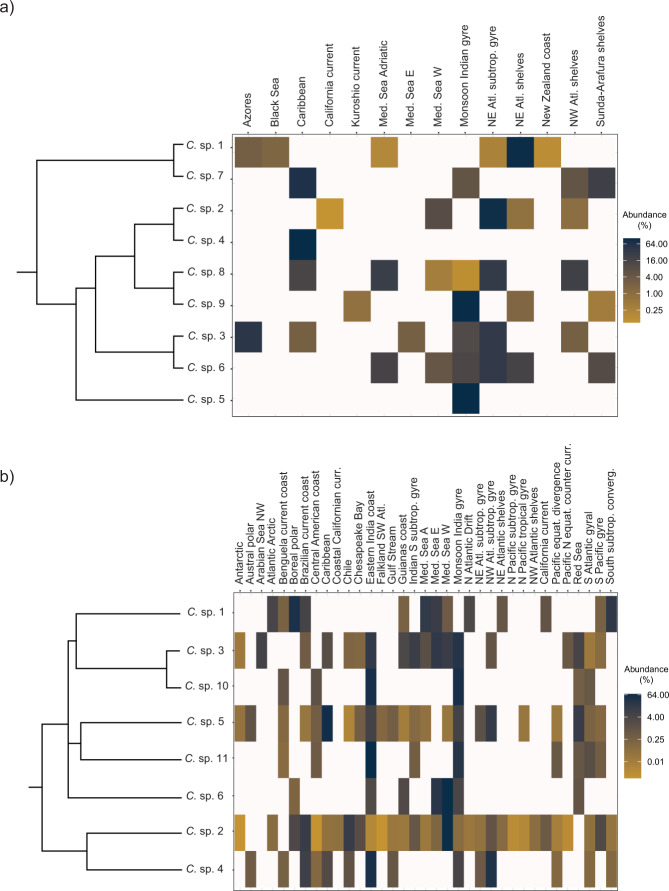
Heatmaps showing the abundance of *C. curvisetus* spp. in each Longhurst’s province. **a** OSD data; **b** OSD-V9 + Tara Oceans data. Data were normalised to the total number of reads for each sample and reported as percentage. Species on the left are ordered according to phylogenetic closeness in the respective networks. For the meaning of the provinces, see ref. [54].

Plots of occurrences gathered from OSD and Tara Oceans metabarcoding data revealed that the species complex is cosmopolitan, occurring in samples in both coastal and open ocean waters at all latitudes in the northern to southern hemispheres (Fig. 2a, b). Yet, the inferred species showed slightly to markedly more restricted distribution patterns (Fig. 4a, b; Supplementary Fig. 3); sp. 1 was found, often abundantly, in the Atlantic, Arctic and temperate provinces, whilst sp. 2 was observed there as well but also all over the tropics and subtropics. Instead, spp. 3, 4 and 5 showed a predominantly warm-temperate to tropical distribution, though the latter two were encountered also near the Antarctic peninsula. Notably, spp. 10 and 11 were more strictly tropical (V9, observable only in Tara Oceans data). The remaining species were encountered less frequently, often at sample sites far apart, e.g. sp. 6 in southern Europe, the Red Sea and scattered along the coasts of the Indian Ocean; sp. 7 in Florida and Singapore; sp. 8 only on the US-East Coast, Panama and the Mediterranean and sp. 9 at a few sites in the tropical Indian Ocean and in Japan (the latter three species observable only in V4, OSD data).

For those species potentially detectable in both datasets (reference sequences including both the V4 and V9), spp. 1 and 6 were well-represented at the OSD sample sites, at least in Europe, but infrequent at the Tara Oceans sites; sp. 2 was observed in both OSD and Tara Oceans sites; and spp. 3, 4 and 5 occurred at many Tara Oceans sites but were rare in the OSD data. Several closely related pairs of species in the V4-tree exhibited distinct distribution ranges (spp. 1 and 7; spp. 2 and 4; spp. 3 and 6; spp. 8 and 9; see Fig. 4a; Supplementary Fig. 2a), and the same was observed between close relatives in the V9 tree (spp. 3 and 10; spp. 5 and 11; spp. 2 and 4; see Fig. 4b; Supplementary Fig. 2b).

## Discussion

### Phylogenetic relationships among taxa belonging to the *C. curvisetus* species complex

Haplotype networks enable delineation of taxa within the *C. curvisetus* species complex and visualisation of the relationships among these taxa. Despite the fact that the global metabarcoding datasets here analysed are different in terms of gene region sequenced and sampling coverage, we retrieve the same number of taxa, including newly inferred ones. This suggests that the molecular information contained in these datasets allows an exhaustive exploration of the complex. In terms of phylogenetic relationships, haplotype networks display relationships better than previously published phylogenetic trees. Indeed, in the trees inferred from the V4- and V9 regions of the 18S rRNA gene [39], as well as in the multigene phylogeny by [53], relationships among the members of this complex are poorly resolved, especially in the tree inferred from the V9 region. The fact that adding more reference barcodes and DNA markers to the phylogenies [53] did not result in any significant improvements in resolution suggests that the approach used to resolve relationships is more important than the number and type of markers analysed. Phylogenies visualise speciation events as dichotomies, whereas haplotype networks can model evolution in a reticulated manner, best fitting cases of recent divergence as may occur in species complexes.

Slight differences in relationships identified in the networks of the V4 and V9 regions of the 18S rRNA gene are likely due to different length of the regions (~384 and ~105 bp, respectively) and are also observed in the V4 and V9 phylogenetic trees in [39]. Furthermore, the reticulations within the networks suggest a weak, but nonetheless existing gene flow between inferred species. The absence of a barcoding gap corroborates that signal, suggesting that the genetic barriers among some members of the complex are incomplete.

The V4 and V9 networks allow proposing hypotheses on putative new species or emerging populations, as also confirmed in the trees obtained using the reference barcodes of *curvisetus* species and the representative sequences of unassigned nodes. Such inference of taxa from metabarcode haplotypes is just the first step of the process; the next step is to try to isolate the target organism in order to link the anonymous sequence to the morphology of the specific taxon. This approach, called ‘reverse taxonomy’ [76] was applied previously in other marine protists and metazoans [14, 77]. In the case of metabarcoding data, the validation of anonymous sequences through isolation of the target organism is supported by abundance tables, which contain information of occurrence, abundance and date for each sampled locality.

### Considerations on Sanger vs. metabarcoding sequencing data

In this work, we have used the accepted barcode for protists (the V4 region of the rRNA gene, [26]) and the V9 region of the rRNA gene to study a cryptic species complex. Instead of a classical, Sanger-based approach of a multitude of geographic strains, we have used metabarcoding datasets (OSD and Tara Oceans), to take advantage of the data available for many sampling localities across the globe. It would have been logistically next to impossible to establish monoclonal strains from all of these sites. As consequence of this choice, we had to work with thousands of sequences. Indeed, differently from a Sanger sequencing approach that provides a single sequence as output (a consensus of all the amplified products), HTS techniques sequence individual molecules. Furthermore, since the 18S gene occurs in hundreds to thousands of copies within the genome, and sometime on multiple chromosomes [78], the number of sequences to handle was even larger. Such rRNA gene copies are expected to be homogenised by concerted evolution over time, but empirical studies suggest that this process is not perfect and multiple, polymorphic copies can persist within the genome [79]. When using environmental samples, 18S rRNA gene copies from different cistrons, chromosomes and individuals are mixed together, precluding the distinction between intra- and inter-specific variability. Using the network approach and simple criteria to assign sequences to a species, we have demonstrated that this is not an issue. All these sequences resulting from the apparent failure of concerted evolution to achieve complete homogenisation, from geographic variability, from PCR and sequencing errors are arranged around the main node in which the ‘dominant haplotype’ is located. All these, i.e. the dominant haplotype and its surrounding peripheral haplotypes contribute to the definition of the species’ overall genetic variation for this marker region. This aspect of concerted evolution suggested by metabarcoding data, as well as the fact that the most abundant haplotype for a specific taxon corresponds to its reference barcode obtained with Sanger sequencing, has been demonstrated recently by [80] in a temporal dataset of Chaetocerotaceae at local scale. In this context, we show that the use of a multi-copy gene is not a disadvantage, but instead, that all these copies contribute to the evaluation of inter- and intra-species variation.

### Eco-evo considerations of the *C. curvisetus* species complex

*C. curvisetus* was originally described from the Kattegat [81] and reported by [82] as a common inhabitant of the Atlantic Ocean and the Baltic Sea, with peaks of abundance in summer and autumn. Hasle and Syvertsen [51] indicated it as a cosmopolitan species mainly found in temperate and warm waters and this was also confirmed by [83]. In Chinese waters, the species has been associated with harmful algal blooms [84, 85], although no production of toxins is known to date. Instead, *C. pseudocurvisetus* is considered by [51] as an inhabitant of warm waters. This finding was partially confirmed by [83], in which the species was found not only in the Mediterranean Sea, the nearby Atlantic Ocean and the Indian Ocean, but also in the North Sea, the latter being quite balmy in high summer.

In general, results of our analysis using OSD and Tara Oceans dataset indicate that the *C. curvisetus* complex is cosmopolitan. Nonetheless, some species show preferences for particular environmental conditions. Furthermore, closely related species often exhibit contrasting geographic distribution patterns mainly related to temperature preferences, especially if they are clearly separated in the network (no reticulation; e.g. spp. 1 and 7). Instead, species connected by reticulate patterns probably still experience gene flow (e.g. spp. 8 and 9) and exhibit more comparable distribution patterns. Despite the fact that the partitioning of ocean regions in Longhurst’s provinces takes into account several biogeochemical parameters, Richter et al. [86] have shown that temperature alone is more correlated to the distribution of larger plankton species (as diatoms) than other environmental parameters.

Other studies involving cryptic species in marine protists have shown similar results. In the genus *Skeletonema*, widely distributed *S. costatum* sensu lato consists of a species complex [5, 87]. Several of its species appear to be widely distributed as well, but within broad climatological boundaries (cool-temperate *S. japonicum*; temperate to tropical *S. tropicum*) whereas others such as *S. grethae* seem to be regional and absent in climatologically comparable regions [13]. More in general, Hasle [88] already noticed that morphologically closely related diatom species are often found in different biogeographic regions. In *Leptocylindrus*, most species were found to be widespread in coastal waters whereas *L. minimus* was restricted to cold waters of the Northern Hemisphere [14, 89]. Similar results were also reported for cryptic species complexes within green algae [90–93]. Our results and the information available in the aforementioned studies indicate that ecological traits are as reliable as phylogenetic data for the circumscription of taxa within a cryptic species complex, and provide a strong support for the formulation of primary species hypotheses.

According to the ‘everything is everywhere’ hypothesis [94], most microbes form populations large enough to migrate efficiently and accumulate mutations that could be beneficial in particular environments [95]. Speciation in the microbial world is therefore expected to involve selection rather than random drift or geographical separation [95]. Diatoms, for example, are believed to exhibit high intra-specific variability, which would be key for their adaptation to different environments [96]. It is possible that different strains of a species already possess beneficial mutations allowing them to adapt to different environments using such intra-specific variability [96]. Once a different environment is reached, some strains would be favoured by natural selection and, over time, accumulate other mutations that will finally differentiate them from the parental population, leading to speciation. In this context, the adaptation to different environments would be the factor triggering speciation in diatoms. Based on the results of this study and data for other protists we conclude that ecological differentiation is likely to facilitate speciation both in allopatric [93, 97] and sympatric [98–100] conditions.

In conclusion, this work shows that the study of cryptic species complexes in marine protists can benefit from the combination of evolutionary approaches and HTS data. First, molecular data from global metabarcoding datasets provide an exhaustive picture of the genetic variability of the cryptic species complex under investigation. Second, the use of phylogenetic networks over trees allows a better visualisation of relationships among closely related taxa, especially when using metabarcoding data from multi-copy genes, where there is no a priori definition of intra- and inter-specific genetic boundaries. Third, the large spatial coverage, including sampling points from different biogeographic regions across world’s oceans, allows the integration of ecological traits in the delimitation of cryptic species. Taken together, all these analyses provide an eco-evolutionary framework for systematic biology assessments of cryptic species.

## Supplementary information

Supplementary Figure 1

Supplementary Figure 2

Supplementary Figure 3

Supplementary Table 1

Supplementary Table 2

Supplementary Table 3

## Data Availability

The fasta reference sequences of the putative new species here identified are available on GenBank at the following accession numbers: MW168796–MW168799. The scripts and input files for generating phylogenetic networks, heatmaps and distribution maps are available on figshare at URL: 10.6084/m9.figshare.13150400. Other Supplementary Information is available for download on the ISME website.
