# Supplementary material for: Resolving cryptic species complexes in marine protists: phylogenetic haplotype networks meet global DNA metabarcoding datasets
Source: ISME J. 2021 Feb 15;15(7):1931–42. doi: 10.1038/s41396-021-00895-0 (PMC8245484; doi:10.1038/s41396-021-00895-0)
Supplement: Supplementary file 1 — Supplementary Figure 1 [file 41396_2021_895_MOESM1_ESM.pdf]

Supplementary Figure 1. Sequence signatures for the members of the *Chaetoceros curvisetus* species complex. (a) V4-18S region; (b) V9-18 region.

a) V4-18S region

```
           ....|....| ....|....| ....|....| ....|....| ....|....| ....|....|
                   5         15         25         35         45         55
C. sp. 1   AGCTCCAATA GCGTATATTA AAGTTGTTGC AGTTAAAAAG CTCGTAGTTG AATTTCTGGC
C. sp. 2   AGCTCCAATA GCGTATATTA AAGTTGTTGC AGTTAAAAAG CTCGTAGTTG AATTTCTGA-
C. sp. 3   AGCTCCAATA GCGTATATTA AAGTTGTTGC AGTTAAAAAG CTCGTAGTTG AATTTCTGGC
C. sp. 4   AGCTCCAATA GCGTATATTA AAGTTGTTGC AGTTAAAAAG CTCGTAGTTG AATTTCTGA-
C. sp. 5   AGCTCCAATA GCGTATATTA AAGTTGTTGC AGTTAAAAAG CTCGTAGTTG AATTTCTGGC
C. sp. 6   AGCTCCAATA GCGTATATTA AAGTTGTTGC AGTTAAAAAG CTCGTAGTTG AATTTCTGGC
C. sp. 7   ---------- ---------- -----GTTGC AGTTAAAAAG CTCGTAGTTG AATTTCTGGC
C. sp. 8   AGCTCCAATA GCGTATATTA AAGTTGTTGC AGTTAAAAAG CTCGTAGTTG AATTTCTGA-
C. sp. 9   AGCTCCAATA GCGTATATTA AAGTTGTTGC AGTTAAAAAG CTCGTAGTTG AATTTCTGA-

           ....|....| ....|....| ....|....| ....|....| ....|....| ....|....|
                  65         75         85         95        105        115
C. sp. 1   TGGAAGGGAG CGGTTCCACA TCTTTGTGGA TACTTGCTCG TCTTCTGCCA TCCGTGGGAC
C. sp. 2   TAGAAGGGAG CGGTTCCGCA TTCTTGCGGA TACTGGCTCG TCTTCCGTCA TCCTTGGGCT
C. sp. 3   TGGAAGGGAG CGGTCCTGCA TTTTTGCAGG TACTTGCTCG TCTTCTGCCA TCCTTGGACT
C. sp. 4   TAGAAGGGAG CGGTTCCGCA TTCTTGCGGA TACTTGCTCG TCTTCCGTCA TCCTTGGGCT
C. sp. 5   TGGAAGGGAG CGGTTCCGCA CTTTTGTGGA TACTTGCTCG TCTTCTGCCA TCCTTGGGCT
C. sp. 6   TGGAAGGGAG CGGTCCCGCA TCTTTGCGGG TACTTGCTCG TCTTCTGCCA TCCTTGGGCT
C. sp. 7   TGGAAGGGAG CGGTTCCACA TCTTTGTGGA CACTTGCTCG TCTTCTGCCA TCTGTGGGAC
C. sp. 8   TAGAAGGGAG CGGTTCCGCA TTCTTGCGGA TACTTGCTCG TCTTCCGTCA TCCTTGGGCT
C. sp. 9   TAGAAGGGAG CGGTTCCGTA TTCTTACGGA TACTTGCTCG TCTTCCGTCA TCCTTGGGCT

           ....|....| ....|....| ....|....| ....|....| ....|....| ....|....|
                 125        135        145        155        165        175
C. sp. 1   TAGTGTCGCC GGTATTAACT TACCGGCGTC TACGATGCCC ACCGTTTACT GTGAGCAAAT
C. sp. 2   TAGTCTCGCT GATATTAACT TATCGGCGTG TACGATGCCC ATCGTTTACT GTGAGCAAAT
C. sp. 3   TAGTCTCACT GATATTAACT TATCGGTGTG TACGATGTCC ATCGTTTACT GTGAGCAAAT
C. sp. 4   TAGTCTCGCT GATATTAACT TATCGGCGTG TACGATGCCC ATCGTTTACT GTGAGCAAAT
C. sp. 5   TAGTCTCGCT GATATTAACT TATCGGTGTG TACGATGCCC ATCGTTTACT GTGAGCAAAT
C. sp. 6   TAGTCTCGCT GATATTAACT TATCGGCGTG TACGATGCCC ATCGTTTACT GTGAGCAAAT
C. sp. 7   TAGTGTCGCC GGTATTAACT TACCGGCGTC TACGATGCCC ATCGTTTACT GTGAGCAAAT
C. sp. 8   TAGTCTCGCT GATATTAACT TATCGGCGTG TACGATGCCC ATCGTTTACT GTGAGCAAAT
C. sp. 9   TAGTCTCGCT GATATTAACT TATCGGCGTG TACGATGCCC ATCGTTTACT GTGAGCAAAT

           ....|....| ....|....| ....|....| ....|....| ....|....| ....|....|
                 185        195        205        215        225        235
C. sp. 1   TAGAGTGTTC AAAGCAGGCT TAT-GCCGTT GAATATATTA GCATGGAATA ATAAGATAGG
C. sp. 2   TAGAGTGTTC AAAGCAGGCA TTTTGCCATT GAATATATTA GCATGGAATA ATAAGATAGG
C. sp. 3   TAGAGTGTTC AAAGCAGGCT TAT-GCCGTT GAATATATTA GCATGGAATA ATAAGATAGG
C. sp. 4   TAGAGTGTTC AAAGCAGGCA TAT-GCCGTT GAATATATTA GCATGGAATA ATAAGATAGG
C. sp. 5   TAGAGTGTTC AAAGCAGGCT TAT-GCCGTT GAATATATTA GCATGGAATA ATAAGATAGG
C. sp. 6   TAGAGTGTTC AAAGCAGGCT TAT-GCCGTT GAATATATTA GCATGGAATA ATAACATAGG
C. sp. 7   TAGAGTGTTC AAAGCAGGCT TAT-GCCGTT GAATATATTA GCATGGAATA ATAAGATAGG
C. sp. 8   TAGAGTGTTC AAAGCAGACT TAT-GTCGTT GAATATATTA GCATGGAATA ATAAGATAGG
C. sp. 9   TAGAGTGTTC AAAGCAGACT TAT-GTCGTT GAATATATTA GCATGGAATA ATAAGATAGG
```

```
              ....|....| ....|....| ....|....| ....|....| ....|....| ....|....|
                   245        255        265        275        285        295
C. sp. 1      ACCTAGTCAC --TATTTTGT TGGTTTGCGT GATTAGATAA TGATTAAGAG GGACAGTTGT
C. sp. 2      ATTTCACGAT TTTATTTTGT TGGTTTGCGT TGTGAAATAA TGATTAAGAG GGACAGTTGT
C. sp. 3      ATCTAGTGAC --TATTTTGT TGGTTTGCGT TATTAGATAA TGATTAAGAG GGACAGTTGT
C. sp. 4      ACTTTTCGAC --TATTTTGT TGGTTTGCGT TGTGAAGTAA TGATTAAGAG GGACAGTTGT
C. sp. 5      ACCTAGTGAC --TATTTTGT TGGTTTGCGT TATTAGGTAA TGATTAAGAG GGACAGTTGT
C. sp. 6      ACCTAGTGAC --TATTTTGT TGGTTTGCGT TATTAGGTAA TGATTAAGAG GGACAGTTGT
C. sp. 7      ACGTAGTGAC --TATTTTGT TGGTTTGTGT CATTACTTAA TGATTAAGAG GGACAGTTGT
C. sp. 8      ACCTAGTGAC --TATTTTGT TGGTTTGCGT TATTAGGTAA TGATTAAGAG GGACAGTTGT
C. sp. 9      ACCTAGTGAC --TATTTTGT TGGTTTGCGT TATTAGGTAA TGATTAAGAG GGACAGTTGT

              ....|....| ....|....| ....|....| ....|....| ....|....| ....|....|
                   305        315        325        335        345        355
C. sp. 1      GGGTACTCGT ATTCAAGTGT CAGAGGTGAA ATTCTTAAAT TACTTGAAGA CGAACGACTG
C. sp. 2      GGGTATTCGT ATTCAGATGT CAGAGGTGAA ATTCTTAGAT TATCGGAAGA CGAACGACTG
C. sp. 3      GGGTATTCGT ATTCAGGTGT AAGAGGTGAA ATTCTTAGAT TATCGGAAGA CGAACGACTG
C. sp. 4      GGGTATTCGT ATTCAGATGT CAGAGGTGAA ATTCTTAGAT TATCGGAAGA CGAACGACTG
C. sp. 5      GGGTATTCGT ATTCAGGTGT AAGAGGTGAA ATTCTTAGAT TATCGGAAGA CGAACGACTG
C. sp. 6      GGGTATTCGT ATTCAGGTGT AAGAGGTGAA ATTCTTAGAT TATCGGAAGA CGAACGACTG
C. sp. 7      GGGTACTCGT ATTCAAGTGT CAGAGGTGAA ATTCTTAGAT TACTTGAAGA CGAACGACTG
C. sp. 8      GGGTATTCGT ATTCAGATGT CAGAGGTGAA ATTCTTAGAT TATCGGAAGA CGAACTACTG
C. sp. 9      GGGTATTCGT ATTCAGATGT CAGAGGTGAA ATTCTTAGAT TATCGGAAGA CGAACGACTG

              ....|....| ....|....| ....
                   365        375
C. sp. 1      CGAAAGCATT TACCAAGGAT GTTT
C. sp. 2      CGAAAGCATT TACCAAGGAT GTTT
C. sp. 3      CGAAAGCATT TACCAAGGAT GTTT
C. sp. 4      CGAAAGCATT TACCAAGGAT GTTT
C. sp. 5      CGAAAGCATT TACCAAGGAT GTTT
C. sp. 6      CGAAAGCATT TACCAAGGAT GTTT
C. sp. 7      CGAAAGCATT TACCAAGGAT GTTT
C. sp. 8      CGAAAGCATT TACCAAGGAT GTTT
C. sp. 9      CGAAAGCATT TACCAAGGAT GTTT
```

(b) V9-18 region

```
                ....|....| ....|....| ....|....| ....|....| ....|....| ....|....|
                    5         15         25         35         45         55
C. sp. 1        GTCGCACCTA CCGATTGAGT GGTCCGGTGA AGGCTCCGGA TTATGGTTAT TGCTTTCATT
C. sp. 2        GTCGCACCTA CCGATTGAGT GACTCGATGA AGGCTCGGGA TTGTGGTTAT TGCTTTCATT
C. sp. 4        GTCGCACCTA CCGATTGAGT GGTTCGATGA AGACTCGGGA TTGAGATTAT TTCTTTCATT
C. sp. 5        GTCGCACCTA CCGATTGAGT GGTCCGGTGA AGACTCCGGA TTATGGTTAT TGCTTTCATT
C. sp. 3        GTCGCACCTA CCGATTGAGT GACCCGGTGA GAATTTCGGA ATGTAGTTGT TGCTTTCATT
C. sp. 6        GTCGCACCTA CCGATTGAGT GGTCCGGTGA AGACTCCGGA TTGTGGTTGT TTCCTTTATT
C. sp. 11       GTCGCACCTA CCGATTGAGT GGTCCGGTGA AGACTCCGGA TTATGGTTGT TCCTTTCACT
C. sp. 10       GTCGCACCTA CCGATTGAGT GACCCGGTGA GAATTTTGGA ATGTGGTCAT TGCTTTCATT

                 ....|....| ....|....| ....|....| ....|....| ....|.
                     65         75         85         95         105
C. sp. 1         AGGAATGACC GTGAGAAGCT GTCTGAACCT TATCACT-AG AGGAAG
C. sp. 2         AGGAATTACT GCGAGAACTT GTCTGAATCT CGCCACTTAG AGGAAG
C. sp. 4         AGGAATTTTT TTTAGAACTT GTCTGAATCT CATCACT-AG AGGCAG
C. sp. 5         AGGAATGATC GTAAGAAGTT GTCTGAACCT TATCACT-AG AGGAAG
C. sp. 3         AGGAATGACC GTGTGAAGCT ATTCGAACCT TGCCACTTAG AGGAAG
C. sp. 6         GGGAATGACC GTGAGAAGTT GTCTGAACCT TATCACT-AG AGGAAG
C. sp. 11        AGGAATGATC GTAAGAAGTT GTCTGAACCT TATCACTTAG AGGAAG
C. sp. 10        AGGAATGACC GTGTGAAGCT ATTCGAACCT TGCCACTTAG AGGAAG
```
