# Supplementary material for: Resolving cryptic species complexes in marine protists: phylogenetic haplotype networks meet global DNA metabarcoding datasets
Source: ISME J. 2021 Feb 15;15(7):1931–42. doi: 10.1038/s41396-021-00895-0 (PMC8245484; doi:10.1038/s41396-021-00895-0)

Supplementary Figure 2. Maximum Likelihood tree of the *C. curvisetus* species complex based on representative sequences of V4 (a) and V9 (b) data. Numbers at the basis of nodes indicate the support to branches after 1 000 bootstrap replicates.

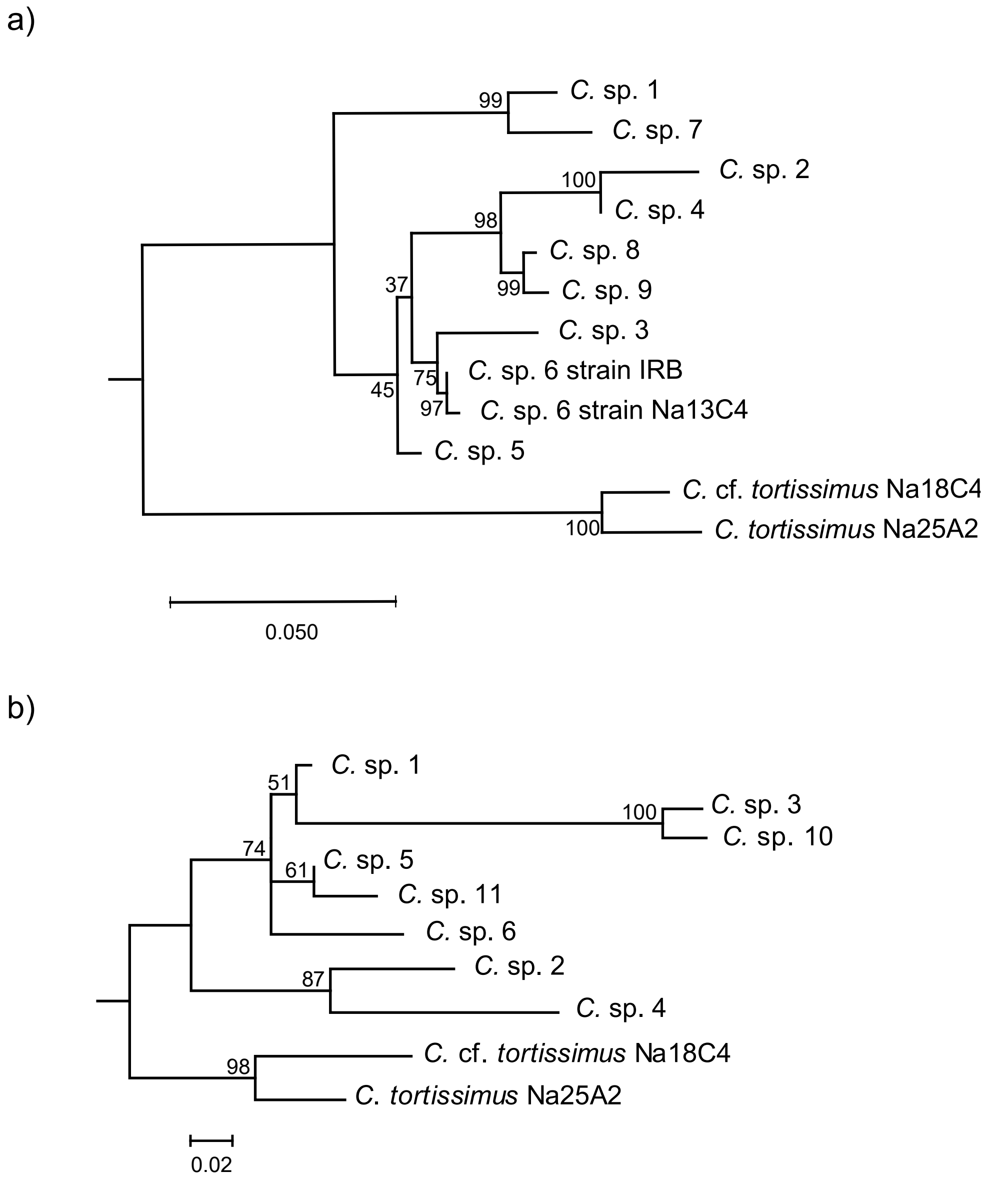

Supplement: Supplementary file 2 — Supplementary Figure 2 [file 41396_2021_895_MOESM2_ESM.pdf]
