# Supplementary material for: Resolving cryptic species complexes in marine protists: phylogenetic haplotype networks meet global DNA metabarcoding datasets
Source: ISME J. 2021 Feb 15;15(7):1931–42. doi: 10.1038/s41396-021-00895-0 (PMC8245484; doi:10.1038/s41396-021-00895-0)

Supplementary Figure 3. Distribution maps of taxa belonging to the *C. curvisetus* species complex here investigated. Light blue dots refer to OSD stations, whilst orange dots to Tara Oceans ones. Numbers in map legend refer to number of reads. Maps are presented showing closely related species next to each other.

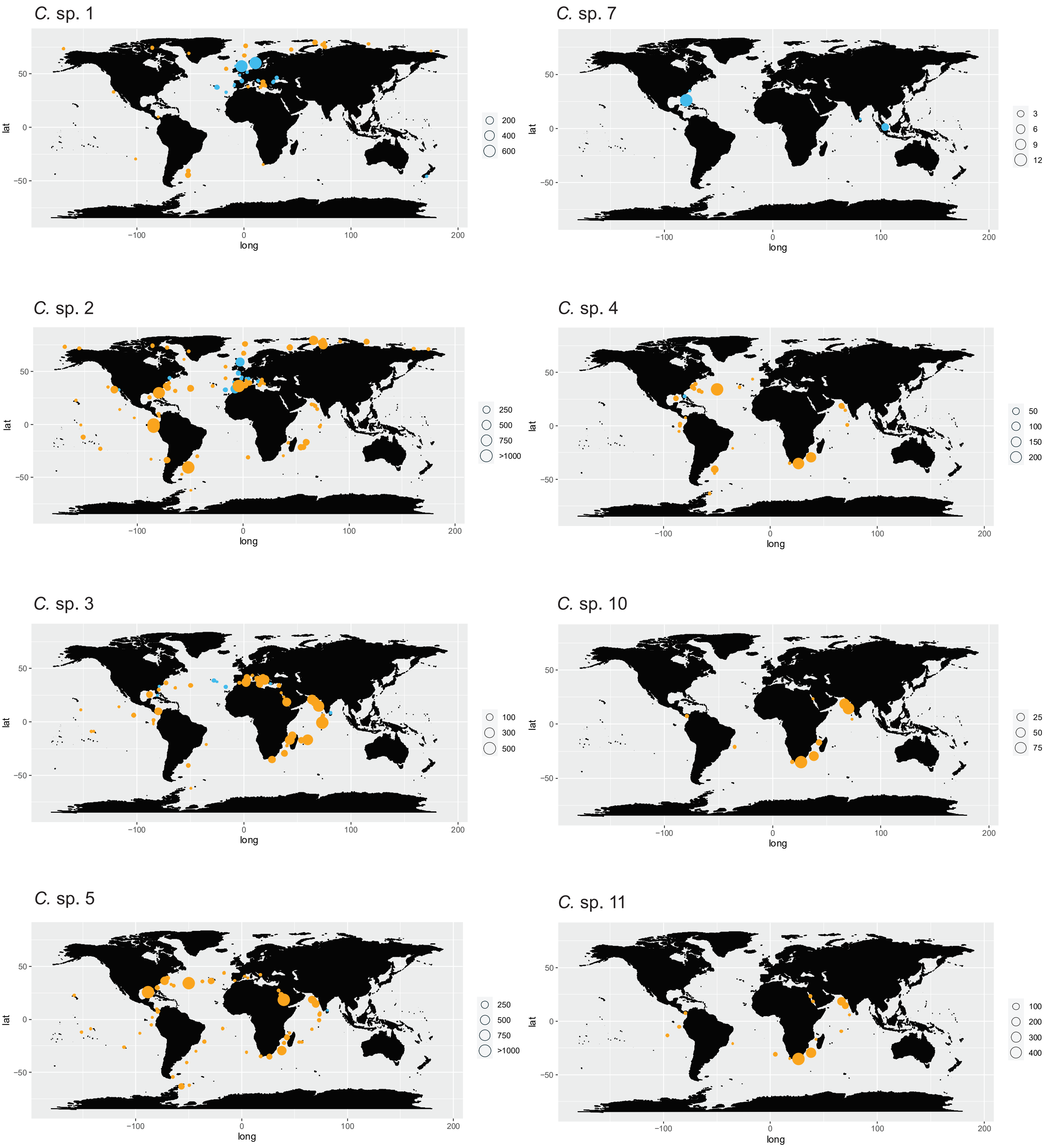

C. sp. 8

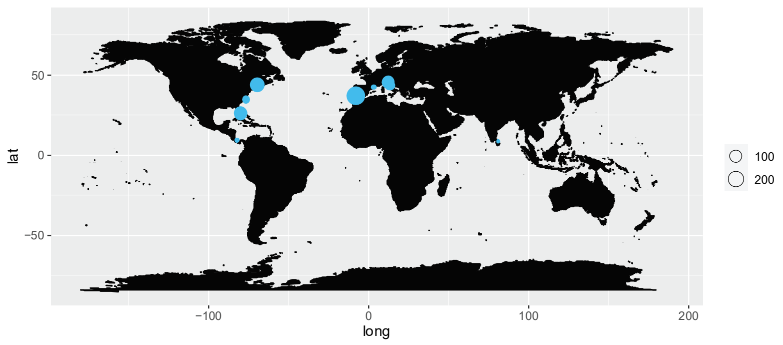

C. sp. 9

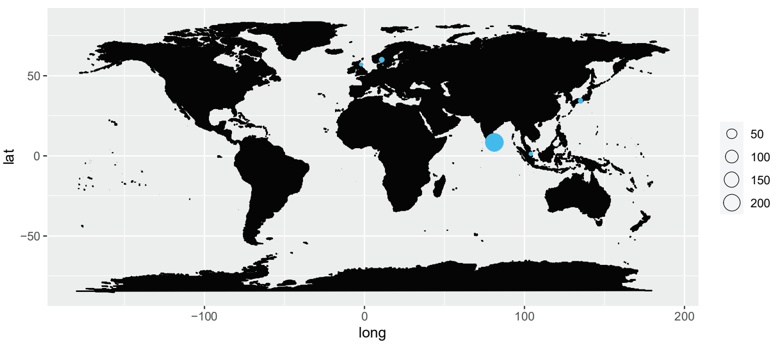

C. sp. 6

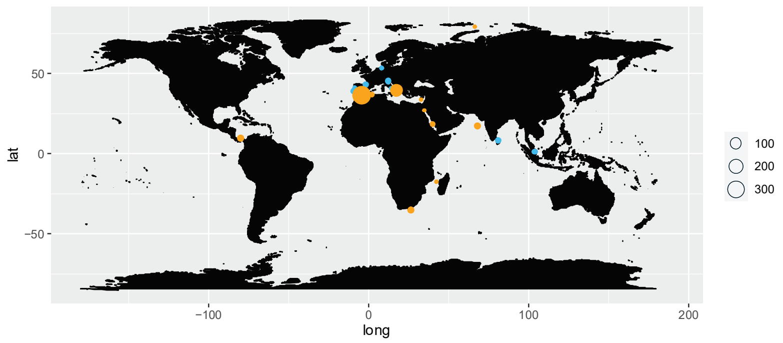

Supplement: Supplementary file 3 — Supplementary Figure 3 [file 41396_2021_895_MOESM3_ESM.pdf]
