# Supplementary material for: Resolving cryptic species complexes in marine protists: phylogenetic haplotype networks meet global DNA metabarcoding datasets
Source: ISME J. 2021 Feb 15;15(7):1931–42. doi: 10.1038/s41396-021-00895-0 (PMC8245484; doi:10.1038/s41396-021-00895-0)
Supplement: Supplementary file 4 — Supplementary Table 1 [file 41396_2021_895_MOESM4_ESM.pdf]

Supplementary Table 1. List of OSD and Tara Oceans sites in which metabarcodes validated as *Chaetoceros curvisetus* spp. were found.

OSD			TARA		
**Station**	**Longitude**	**Latitude**	**Station**	**Longitude**	**Latitude**
OSD2	-3,938	48,778	TARA_004	-6,553	36,563
OSD4	14.25	40,808	TARA_005	-4,406	36,030
OSD5	24.99	35,661	TARA_006	-4,251	36,529
OSD6	2.8	41,667	TARA_007	1,948	37,031
OSD13	27,909	43,176	TARA_008	3,966	38,011
OSD14	3.15	42.49	TARA_009	5,820	39,112
OSD22	5,175	43,226	TARA_010	2,865	40,668
OSD24	-2.88	35,193	TARA_011	2,798	41,666
OSD26	-5.75	35.82	TARA_012	7,899	43,348
OSD29	-80,283	27,469	TARA_014	12,858	39,902
OSD37	-80,093	26,103	TARA_016	15,454	37,398
OSD38	-80,784	24,745	TARA_017	14,306	36,258
OSD43	-117,257	32,867	TARA_018	14,288	35,756
OSD50	-1,925	43,333	TARA_019	13,865	34,216
OSD51	-82,266	9,348	TARA_020	14,973	34,451
OSD54	-69,641	43,844	TARA_022	17,400	39,729
OSD55	-69,578	43.86	TARA_023	17,729	42,176
OSD58	-76,671	34,718	TARA_024	17,956	42,457
OSD60	-79,168	33,323	TARA_025	19,421	39,333
OSD64	30,776	46,442	TARA_026	20,188	38,431
OSD69	12.26	45,457	TARA_030	32,789	33,929
OSD70	12,438	45,414	TARA_031	34,819	27,151
OSD71	170,771	-45,744	TARA_032	37,254	23,391
OSD74	-8,667	41,142	TARA_033	38,218	22,057
OSD76	12,935	43,948	TARA_034	39,884	18,445
OSD77	13,073	43,851	TARA_036	63,524	20,824
OSD78	13,595	43.57	TARA_038	64,576	19,017
OSD81	-7,973	37,005	TARA_039	66,463	18,647
OSD91	-9,037	32,747	TARA_040	67,984	17,500
OSD92	-7,701	33,584	TARA_041	70,011	14,582
OSD94	-2,215	35,086	TARA_042	73,919	5,992
OSD95	103,917	1,268	TARA_043	73,489	4,660
OSD97	-28,602	38.53	TARA_044	71,520	2,806
OSD98	-28.13	38.64	TARA_045	71,710	0.941
OSD101	-16,711	32,742	TARA_046	73,162	-0.659
OSD102	-16.91	32,646	TARA_047	72,164	-2,042
OSD107	-9.38	39.14	TARA_048	66,320	-9,408
OSD108	-8,966	38,757	TARA_049	59,504	-16,808
OSD109	-9,012	38,677	TARA_050	56,795	-21,476
OSD110	-8,869	40,145	TARA_051	54,283	-21,476
OSD115	-9,385	39,134	TARA_052	53,508	-17,023
OSD116	-9,219	39,415	TARA_053	46,923	-13,070
OSD117	-7,504	37,167	TARA_054	45,226	-12,813
OSD124	135,121	34,324	TARA_057	42,742	-17,026

OSD131	27,401	42,245
OSD145	3,119	51,361
OSD147	81,052	8,522
OSD148	8,149	53,581
OSD153	-7,973	36,998
OSD154	-1,167	44,667
OSD155	10,599	59,816
OSD156	10.72	59.9
OSD157	10,628	59,622
OSD158	-25.19	37,433
OSD159	-4,552	48,359
OSD162	-2,103	56,963
OSD163	-2,973	58,957
OSD166	2.9	43,433
OSD173	3.14	51,441
OSD177	2,702	51,186

TARA_058	42,320	-17,455
TARA_062	40,182	-22,339
TARA_064	37,929	-29,508
TARA_065	26,334	-35,226
TARA_066	18,016	-34,905
TARA_068	4,620	-31,039
TARA_072	-18,006	-8,691
TARA_076	-35,231	-21,029
TARA_078	-43,323	-30,158
TARA_080	-51,952	-40,698
TARA_081	-52,214	-44,497
TARA_082	-58,012	-47,165
TARA_083	-65,023	-54,418
TARA_085	-49,503	-62,176
TARA_088	-56,806	-63,386
TARA_092	-71,977	-33,697
TARA_094	-87,093	-32,765
TARA_096	-101,268	-29,655
TARA_098	-110,992	-26,261
TARA_100	-96,283	-13,162
TARA_102	-85,270	-5,218
TARA_106	-84,620	0.037
TARA_109	-84,545	1,800
TARA_110	-84,616	-1,913
TARA_113	-134,920	-23,114
TARA_114	-134,912	-23,130
TARA_115	-134,931	-23,216
TARA_116	-134,931	-23,217
TARA_118	-135,009	-23,129
TARA_120	-134,912	-23,012
TARA_123	-140,304	-8,878
TARA_125	-142,610	-8,890
TARA_126	-151,208	-11,975
TARA_128	-153,305	-0.469
TARA_130	-152,462	11,265
TARA_131	-158,052	22,746
TARA_133	-127,750	35,343
TARA_134	-121,986	32,667
TARA_135	-121,832	32,983
TARA_137	-116,699	14,161
TARA_138	-103,017	6,216
TARA_140	-79,312	7,471
TARA_141	-80,086	9,834
TARA_142	-88,417	25,602
TARA_143	-79,682	29,885
TARA_144	-72,815	36,369
TARA_145	-70,076	39,163
TARA_146	-71,248	34,731

TARA_147	-66,533	32,954
TARA_148	-64,145	31,782
TARA_149	-49,840	34,098
TARA_150	-37,102	35,800
TARA_151	-28,801	36,194
TARA_152	-16,662	43,668
TARA_153	-16,564	44,034
TARA_155	-16,755	54,597
TARA_158	0.374	67,193
TARA_163	1,689	76,078
TARA_168	44,126	72,582
TARA_173	75,345	78,939
TARA_175	66,384	79,343
TARA_178	73,235	77,234
TARA_180	75,459	75,172
TARA_188	91,725	78,304
TARA_189	116,482	78,022
TARA_191	160,961	71,549
TARA_193	174,901	71,115
TARA_194	-168,518	73,336
TARA_196	-154,934	71,895
TARA_201	-85,729	74,329
TARA_205	-71,952	72,423
TARA_208	-51,578	69,107
TARA_210	-55,985	61,544
