# Supplementary material for: Resolving cryptic species complexes in marine protists: phylogenetic haplotype networks meet global DNA metabarcoding datasets
Source: ISME J. 2021 Feb 15;15(7):1931–42. doi: 10.1038/s41396-021-00895-0 (PMC8245484; doi:10.1038/s41396-021-00895-0)
Supplement: Supplementary file 6 — Supplementary Table 3 [file 41396_2021_895_MOESM6_ESM.pdf]

Supplementary Table 3. Average evolutionary distances over sequence pairs within species. Numbers of base substitutions per site were averaged over all sequence pairs within each group. Distances are according to Jukes-Cantor. A dash indicates species absence.

	**Distances V4 region**			**Distances V9 region**		
**Species**	**Mean**	**Min**	**Max**	**Mean**	**Min**	**Max**
C. sp. 1	0.009	0.000	0.054	0.013	0.000	0.029
C. sp. 2	0.008	0.000	0.035	0.017	0.000	0.049
C. sp. 3	0.007	0.000	0.021	0.013	0.000	0.029
C. sp. 4	0.000	0.000	0.000	0.012	0.000	0.029
C. sp. 5	0.010	0.003	0.016	0.016	0.000	0.039
C. sp. 6	0.010	0.003	0.035	0.014	0.000	0.029
C. sp. 7	0.013	0.003	0.105	-	-	-
C. sp. 8	0.008	0.000	0.027	-	-	-
C. sp. 9	0.007	0.000	0.098	-	-	-
C. sp. 10	-	-	-	0.000	0.000	0.000
C. sp. 11	-	-	-	0.016	0.000	0.039
